# Clinical significance of circulating tumor cells and metabolic signatures in lung cancer after surgical removal

**DOI:** 10.1186/s12967-020-02401-0

**Published:** 2020-06-17

**Authors:** Dawei Yang, Xiaofang Yang, Yang Li, Peige Zhao, Rao Fu, Tianying Ren, Ping Hu, Yaping Wu, Hongjun Yang, Na Guo

**Affiliations:** 1 Zhong Yuan Academy of Biological Medicine, Liaocheng People’s Hospital, Liaocheng, 252000 People’s Republic of China; 2 Experimental Research Center, China Academy of Chinese Medical Sciences, Beijing, 100700 People’s Republic of China; 3 Department of Respiratory Medicine, Liaocheng People’s Hospital, Liaocheng, 252000 People’s Republic of China; 4 Institute of Chinese Materia Medica, China Academy of Chinese Medical Sciences, Beijing, 100700 People’s Republic of China; 5 State Key Laboratory of Generic Manufacture Technology of Traditional Chinese Medicine, Lunan Pharmaceutical Group Co. Ltd., Shandong, 276006 People’s Republic of China

**Keywords:** Lung cancer, Circulating tumor cells (CTC), Metabolomics, Negative enrichment-fluorescence in situ hybridization (NE-FISH), Metabolic pathway analysis

## Abstract

**Background:**

Lung cancer (LC) remains the deadliest form of cancer globally. While surgery remains the optimal treatment strategy for individuals with early-stage LC, what the metabolic consequences are of such surgical intervention remains uncertain.

**Methods:**

Negative enrichment-fluorescence in situ hybridization (NE-FISH) was used in an effort to detect circulating tumor cells (CTCs) in pre- and post-surgery peripheral blood samples from 51 LC patients. In addition, targeted metabolomics analyses, multivariate statistical analyses, and pathway analyses were used to explore surgery-associated metabolic changes.

**Results:**

LC patients had significantly higher CTC counts relative to healthy controls with 66.67% of LC patients having at least 1 detected CTC before surgery. CTC counts were associated with clinical outcomes following surgery. In a targeted metabolomics analysis, we detected 34 amino acids, 147 lipids, and 24 fatty acids. When comparing LC patients before and after surgery to control patients, metabolic shifts were detected via PLS-DA and pathway analysis. Further surgery-associated metabolic changes were identified when comparing LA (LC patients after surgery) and LB (LC patients before surgery) groups. We identified SM 42:4, Ser, Sar, Gln, and LPC 18:0 for inclusion in a biomarker panel for early-stage LC detection based upon an AUC of 0.965 (95% CI 0.900–1.000). This analysis revealed that SM 42:2, SM 35:1, PC (16:0/14:0), PC (14:0/16:1), Cer (d18:1/24:1), and SM 38:3 may offer diagnostic and prognostic benefits in LC.

**Conclusions:**

These findings suggest that CTC detection and plasma metabolite profiling may be an effective means of diagnosing early-stage LC and identifying patients at risk for disease recurrence.

## Introduction

Lung cancer (LC) is among the most common forms of cancer globally, with 2.1 million new cases and 1.8 million LC-related deaths in 2018 alone [1]. LC has a 5-year survival rate of < 20%. This poor prognosis is attributable in part to the fact that many patients are only diagnosed when the disease is at an advanced stage, with metastases often being present. Standard treatments for LC at present include radiotherapy, chemotherapy, surgical resection, and targeted therapeutic interventions [2, 3]. Surgical treatment of patients with early-stage (stage I/II) is associated with good long-term outcomes [4, 5]. Surgery is also important for treating individuals with more advanced (stage III/IV) LC [6]. Recent surgical innovations such as video-assisted thoracoscopic surgery have also decreased the invasivity of these intervention protocols, thus allowing more patients to be candidates for surgical treatment [7].

A number of different cancer-associated materials can be detected in circulating peripheral blood, including tumor-derived exosomes, circulating tumor DNA (ctDNA), and circulating tumor cells (CTCs) [8]. Specific cancer-derived proteins including carcinoembryonic antigen (CEA) and alpha-fetoprotein (AFP) are similarly detectable, as well as circulating metabolites. Measurement of these markers can offer insight into disease progression in both primary tumor and metastatic compartments, and can also be used as a means of screening for and diagnosing early-stage cancers.

CTCs are released from primary or metastatic tumors into circulation [9]. Although these cells are extremely rare in most cancer patients, recent improvements in CTC capture, enrichment, and detection technologies have significantly improved the clinical feasibility of analyzing these cells to monitor tumor progression and therapeutic responsiveness [9–11]. CTC levels have been shown to be independently predictive of patient outcomes in patients with both non-small cell lung cancer (NSCLC) [12, 13] and small cell lung cancer (SCLC) [14, 15]. Cancers can further be monitored in real time via single-cell-based analyses of these CTCs, thus offering a more direct insight into the metastatic progression of a given patient’s cancer [16–18]. CTC detection may thus allow for the identification of those LC patients with a higher risk of disease recurrence, potentially allowing clinicians to determine which patients are most likely to benefit from adjuvant treatment following surgery.

Surgery is likely to alter metabolite profiles in LC patients such that metabolomic studies may allow for a more in-depth understanding of these operation-associated shifts. Metabolomic studies allow for the simultaneous analysis of thousands of different endogenous metabolites in a given biological sample. Previous studies have employed metabolomic approaches in studies of cancers of the lung, breast, prostate, bladder, and other tissues [19–21]. These metabolomic approaches have allowed for the identification of cancer-associated biomarkers and/or biomarkers associated with therapeutic responsiveness, thus allowing for the monitoring of these biomarkers in order to better understand disease progression [22]. Previous studies have sought to identify metabolite biomarkers associated with LC patient outcomes [23]. How surgical tumor resection affects these metabolite profiles, however, remains uncertain.

Metabolite profiling can be sensitively conducted using an ultra-performance liquid chromatography/mass spectrometry (UPLC/MS) platform [24, 25]. In the present study, we utilized ultra-performance liquid chromatography with quadrupole time-of-flight mass spectrometry (UPLC-QTOF/MS) and ultra-performance liquid chromatography-tandem mass spectrometry (UPLC-MS/MS) approaches together with multivariate statistical approaches in order to explore shifts in LC patient metabolomic serum profiles in response to surgical intervention. We additionally quantified CTC frequencies in these patients before and after surgery and explored the prognostic relevance of metabolic and CTC signatures in this patient population.

## Materials and methods

### Chemicals and materials

MS-grade methanol, formic acid, acetic acid and acetonitrile were purchased from Fisher scientific. Ultra-pure water (18.2 MΩ) was prepared with a Milli-Q water purification system (Millipore, Bedford, MA, USA). Glycine (Gly), β-alanine (β-Ala), sarcosine (Sar), l-alanine (Ala), l-serine (Ser), l-proline (Pro), l-valine (Val), l-threonine (Thr), l-cysteine (Cys), taurine (Tau), l-leucine (Leu), γ-aminobutyric acid (GABA), l-isoleucine (Ile), trans-4-hydroxy-l-proline (t-Hyp), l-asparagine (Asn), l-aspartic Acid (Asp), l-ornithine (Orn), l-lysine (Lys), l-glutamic acid (Glu), l-methionine (Met), 1-methyl-l-histidine, 3-methyl-l-histidine, l-2-aminoadipic acid, l-phenylalanine (Phe), l-arginine (Arg), l-citrulline (Cit), l-tyrosine (Tyr), l-tryptophan (Trp), l-carnosine, folic acid (FA), kynurenic acid, pipecolinic acid were purchased from Sigma-Aldrich company (St. Louis, MO, USA); l-histidine (His) and glutamine (Gln) were purchased from TCI (Develoment Co., Ltd, Shanghai, China); Phenylalanine-d5 (Internal standard, IS) was purchased from Toronto Research Chemicals (YTO, Canada). The categories of lysophosphatidylcholine (LPC), phosphatidylcholine (PC), lysophosphatidylethanolamine (LPE), phosphatidylethanolamine (PE), sphingomyelin (SM), ceramide (Cer), were purchased from Avanti Polar Lipids company (Alabaster, AL, USA); triacylglycerol (TAG), free fatty Acid were purchased from Larodan company (Stockholm, Sweden). ISs of PC (19:0/19:0), LPC (19:0/0:0), PE (12:0/13:0), SM(d18:1/12:0), Cer (d18:1/17:0) were purchased from Avanti Polar Lipids company (Alabaster, AL, USA), TAG (15:0/15:0/15:0), FFA (C19:0), d3-FFA(C16:0) were purchased from Larodan company (Stockholm, Sweden). All other chemicals and reagents used were of analytical grade.

### Study population

Patients that presented to the Liaocheng People’s Hospital LC clinic with suspected LC from September 2016–January 2018 were included in the present study. A total of 51 paired pre- and post-operative serum samples were collected from patients that had provided written informed consent to participate (Table 1). All patients were newly diagnosed and untreated. All cancer patients were confirmed by histopathological diagnosis. We excluded patients who recently received radiotherapy, chemotherapy or immunotherapy, and had non-pulmonary primary tumor. Patients with benign diseases were diagnosed by imaging, serum tests, and histopathology. Criteria for healthy donors included non-smoking; non-alcoholic; no family history of cancer; no tumors or other disease history; normal heart, liver, lung and brain; and normal results on routine tests for blood, urine, feces, tumor markers, renal and liver function, chest X-ray and electrocardiography. Following tumor resection, histopathological confirmation of LC diagnosis was made, and tumor stage and clinicohistopathological characteristics were analyzed. As a control group, 51 age- and sex-matched healthy volunteers were recruited. The Ethics Committee of Liaocheng People’s Hospital approved this study, which was conducted in a manner consistent with the Declaration of Helsinki.

**Table 1:** The relationship between patient demographics, clinical characteristics, and CTC levels

Characteristics	*n*	Proportion (%)	CTC < 1		CTC ≥ 1		*P*
			*n*	Proportion (%)	*n*	Proportion (%)	
Gender							
Male	29	56.86	7	24.14	22	75.86	0.104
Female	22	43.14	10	45.45	12	54.55	
Age							
≥ 60	35	68.63	10	28.57	25	71.43	0.602
< 60	16	31.37	7	43.75	9	56.25	
Histology							
Adenocarcinoma	31	60.78	12	38.71	19	61.29	0.904
Squamous	16	31.37	4	25	12	75	
SCLC	4	7.84	1	25	3	75	
Distant metastasis							
M0	49	96.08	16	32.65	33	67.35	0.306
M1	2	3.92	1	50	1	50	
Tumor depth							
T1	29	56.86	14	48.28	15	51.72	0.141
T2	13	25.49	3	23.08	10	76.92	
T3	5	9.80	0	0	5	100	
T4	4	7.84	0	0	4	100	
Lymph node metastasis							
Yes	18	35.29	0	0	18	100	0.003
No	33	64.71	17	51.52	16	48.48	
TNM stage (UIUC)							
I	29	56.86	16	55.17	13	44.83	0.001
II	10	19.61	0	0	10	100	
III	10	19.61	0	0	10	100	
IV	2	3.92	1	50	1	50	

### Sample preparation

Preoperative serum samples were collected from LC patients on the morning following hospitalization, while postoperative serum samples were collected on the last morning prior to discharge for each patient (7 days post-operation). For all sample collections, peripheral blood (~ 3.2 mL) was collected in a Citrate-dextrose solution (ACD) Vacutainer tube (BD, NJ, USA), and samples were stored at room temperature (RT) prior to analysis for CTC levels. For metabolomics assays, an additional 2 mL of serum was collected for analysis, with control samples being collected contemporaneously.

### Clinical effect criteria

RECIST (Response Evaluation Criteria in Solid Tumors) v1.1 was used for the evaluation of clinical responses. Responses were classified based upon tumor diameter and axillary lymph node status as follows: complete response (CR), stable disease (SD), partial response (PR), or progressive disease (PD). Clinical efficacy was considered to have been achieved when PR or CR was diagnosed. For analysis purposes, patients with CR or PR were incorporated into one analysis group (CR + PR) while patients with SD or PD were incorporated into a second group (SD + PD). Patients underwent follow-up through October 31, 2019, via phone call and outpatient visits. The time between surgery and the last diagnosis of recurrence was 21–37 months. A total of 14 LC patients suffered from disease recurrence, while 31 did not suffer from recurrence and 6 did not have sufficient available clinical information to determine whether disease had recurred.

### CTC detection

**Fig. 1:**
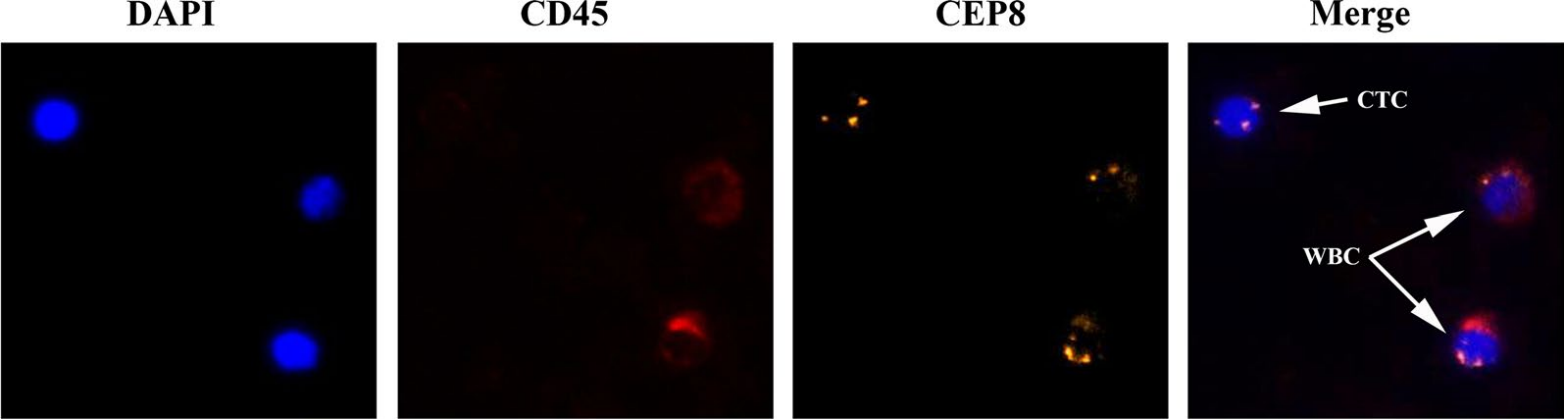
Sample CTC graphs. *CEP* centromere probe, *CTCs* circulating tumor cells, *WBC* white blood cells

Negative enrichment fluorescence in situ hybridization (NE-FISH) was used to detect CTCs as in our previous studies [26, 27]. Briefly, patient blood samples were washed and RBCs were lysed with the CS1 and CS2 buffers, respectively, followed by resuspension in a solution containing magnetic particles bound to anti-CD45 (Cyttel Biosciences INC., Jiangsu, China). Gradient centrifugation was then performed using the CS3 buffer (Cyttel) to separate samples, and a magnetic stand was used to collect the CTC-containing sample. This sample was then fixed and spread onto a slide, which was then dried for analysis. FISH was then performed using the chromosome centromere probe (CEP) 8 + 17, with DAPI used to stain cell nuclei and AF594-conjugated anti-CD45 being used to stain cells. Slides were then imaged via microscopy (BX63, Olympus), and an IMSTAR high content screening device equipped with the PathfinderTM software (IMSTAR S.A., Paris, France) was used for image analysis. CTCs were cells that were found to be DAPI+/CD45−/CEP+ (Fig. 1).

### UPLC-MS/MS

Amino acids, lipids, and fatty acids were analyzed with a UPLC system (Waters ACQUITY) that contained a quaternary pump, an autosampler, a degasser, and a Xevo TQ-S micro mass spectrometer with an ESI ionization source (Waters, MA, USA). The amino acid analysis was conducted following sample separation with a Waters ACQUITY UPLC BEH Amide column (2.1 mm × 100 mm, 1.7 μm; 0.3 mL/min flow rate). For this separation, the mobile phases used were 0.1% and 0.2% formic acid (A and B, respectively). A linear gradient was used for separation as follows: 0–0.5 min, 15% B; 0.5–5.5 min, 15–20% B; 5.5–12.5 min, 20–40% B; 12.5–13 min, 40–15% B; and 13–15 min, 15% B. An initial 5 μL injection volume was used, and the column was warmed to 40 °C. Between each sample injection, the column was washed once twice using a weak 90% acetonitrile solution and a strong 10% acetonitrile solution.

A Waters ACQUITY UPLC BEH C8 column (2.1 × 100 mm, 1.7 μm; 0.26 mL/min flow rate) was used for analyses of fatty acids and lipids. For these separations, 60% acetonitrile in water containing 5 mM ammonium formate and 90% isopropanol in acetonitrile containing 5 mM ammonium formate served as the mobile phases (A and B, respectively). A linear gradient was used for separation as follows: 0–1.0 min, 100% A; 1.0–2.0 min, 100–70% A; 2.0–12.0 min, 70–30% A; 12.0–12.5 min, 30–5% A; 12.5–13.0 min, 5–0%A; 13.0–14.0 min, 0% A; 14.0–14.1 min, 0–100% A and 13–15 min, 100% A. Injection volumes in positive and negative ion mode were 1 μL and 2 μL, respectively, and the column was warmed to 55 °C.

Positive and negative ion modes and multiple reaction monitoring (MRM) were employed during micro mass spectrometer (Xevo TQ-S) operation, with data being acquired and processed using the Waters TargetLynx software.

### Data analysis

SPSS (v17, SPSS Inc, IL, USA) was used for all statistical testing. Receiver operating characteristic (ROC) curves were used to explore the diagnostic sensitivity and specificity of CTCs for LC diagnosis. Data were compared via non-parametric Wilcoxon’s test and Kruskal–Wallis H test.

Metaboanalyst [28, 29] was used for multivariate statistical analyses of metabolomics data, including partial least squares discriminant analysis (PLS-DA), biomarker analyses, and pathway analyses. The *R*^2^ and *Q*^2^ statistics were utilized to assess PLS-DA model quality, while its reliability was assessed via a permutation test owing to the potential for this model to overestimate separation performance [28, 30]. Univariate analysis, including Student’s t-test and Analysis of Variance (ANOVA), were used in the study. Binary logistic regression was carried out for variable selection in the model.

For a pathway analysis of differentially expressed metabolites, pathway modules and pathway topology analyses were used to best identify key biological pathways linked to the observed changes in metabolite levels. The resultant data show both P-values in the pathway enrichment analysis (y-axis) and the pathway impact values from pathway topology analysis (x-axis), with red being used to identify the most affected pathways.

## Results

### CTC detection in LC and control patients

**Fig. 2:**
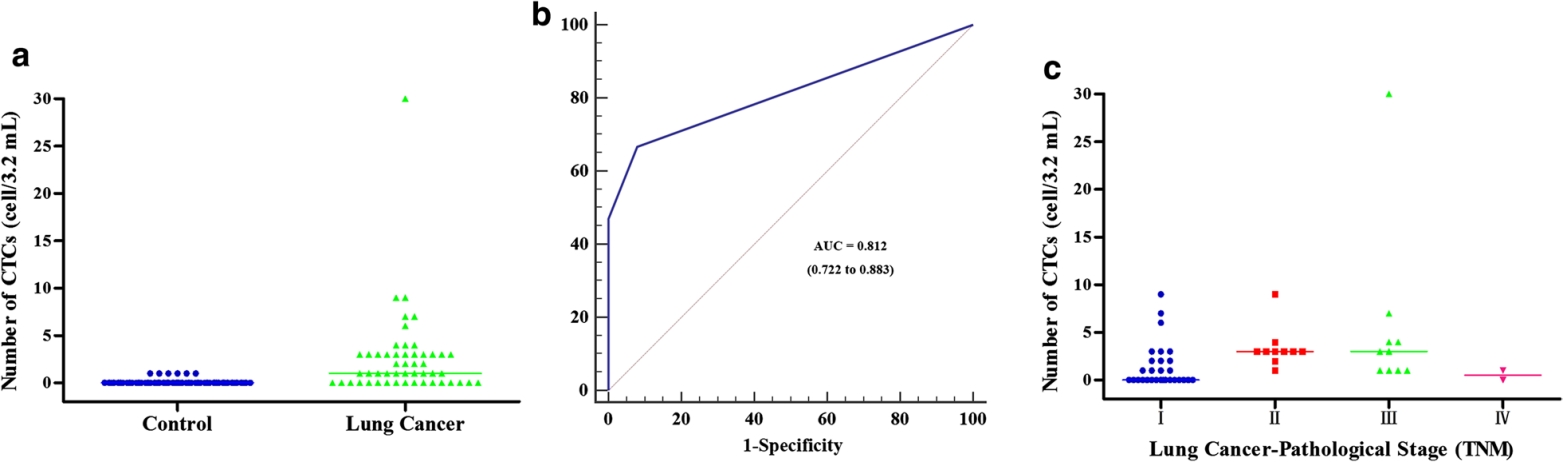
CTC numbers in lung cancer patients and controls. **a** CTC distribution in controls and lung cancer patients. **b** ROC curves used for CTC cutoff value determinations. **c** CTC distributions in patients with different pathological stages of disease

We have previously used an NE-FISH-based approach to detect CTCs in lung cancer patient peripheral blood samples. In this study, we detected ≥ 1 CTC in 34/51 (66.67%) patients prior to surgery (Table 1). Total CTC numbers were from 1 to 30 (mean = 2.57), with significantly more CTCs being present in LC patients relative to controls (*P* < 0.001, Fig. 2a). An ROC curve was employed to assess the diagnostic utility of CTCs in LC, revealing them to have sensitivity and specificity values of 66.67% and 92.16%, respectively. This analysis revealed that a cut-off of 0.5 CTCs/3.2 mL of blood was sufficient for LC diagnosis (AUC = 0.812, 95% CI 0.722–0.883, *P* < 0.0001) (Fig. 2b). As such, we utilized a 1 CTC cut-off value for LC diagnosis. The CTC positivity rates in SCLC patients were 75%, while in NSCLC patients with adenocarcinomas and squamous cell carcinomas they were 61.29% and 75%, respectively. TNM stages and lymph node status differed significantly as a function of CTC counts, with all patients with lymph node metastasis having detectable CTCs (Table 1).

After surgery, CTCs were detectable in 34/51 LC patients, with CTC number haven risen in 18 patients, fallen in 21 patients, and remained unchanged in 13 patients.

### Targeted metabolomics analyses

We next explored the levels of specific lipids, fatty acids, and amino acids in LC patient serum through a targeted metabolomics analysis. During the analytical process, quality control samples (QC) were regularly run to ensure result reproducibility, with peak area RSD for these QC samples being < 15% consistent with good stability and reproducibility. A total of 34 underivatized amino acids were assessed in patient and control serum samples [31]. Direct infusion mode was used for sample introduction into the mass spectrometer. Additional file 1: Table S1 compiles information pertaining to the precursor ions, product ions, and collisions for lipid and fatty acid samples. In total, 24 fatty acids and 147 lipids were detected in this analysis.

### Profiling of global metabolic changes in LC patients following surgical intervention

**Fig. 3:**
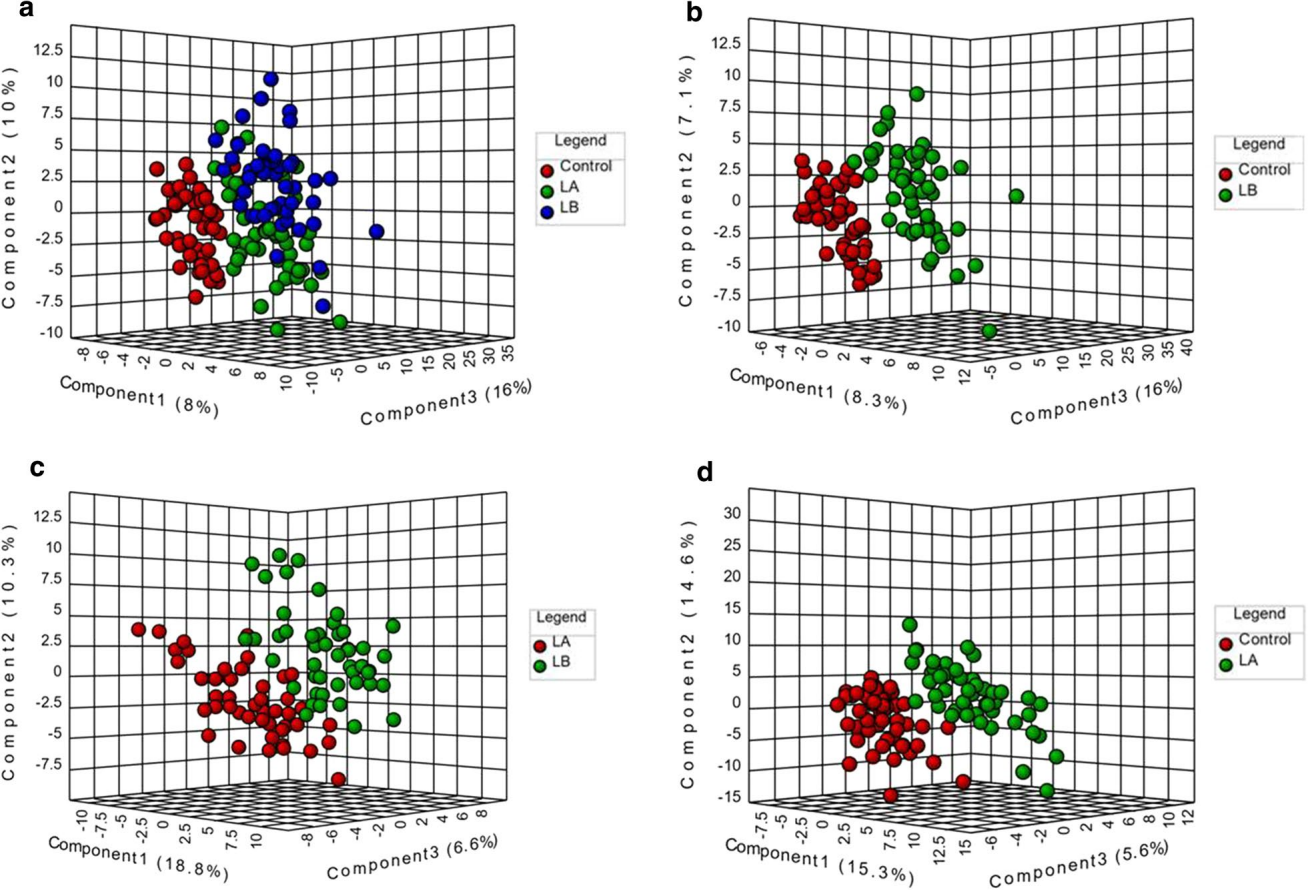
Score plots for PLS-DA analysis model of metabolomics data the control, LA, and LB groups. **a** PLS-DA results in all groups; **b** PLS-DA results in control and LB groups; **c** PLS-DA results in the LA and LB groups; **d** PLS-DA results in control and LA groups

We employed a PLS-DA approach in order to explore the metabolic differences between the control, LA (LC patients after surgery), and LB (LC patients before surgery) groups (Fig. 3). A permutation test revealed that the B/W ratio of the original classes (red arrow) different significantly from the permuted data distribution, consistent with reliable cross-validation (*P* < 0.05, Additional file 1: Figure S1). This PLS-DA approach achieved satisfactory classification. Control patients exhibited a significantly distinct metabolic profile, whereas that of the LA and LB groups overlapped slightly (Fig. 3a). However, good separation was achieved between these three groups (Fig. 3b–d). Pre-and post-surgical LA, LB, and control groups could be distinguished on the basis of amino acids and lipid profiles, consisting with significant surgery-related metabolic shifts being present in LC patients and controls (Fig. 3b, d), and there were additional changes between groups LA and LB as a result of surgery.

### Differentially abundant metabolite patterns

A univariate statistical analysis revealed significant changes in lipids, fatty acids and amino acids between the LA, LB, and control groups (Table 2). When comparing the control and LB groups, levels of Cer, PC, SM lipids were higher in the LB group, while levels of LPC, LPE, and TAG were higher in controls. Similarly, LB group samples exhibited higher levels of the amino acids Asn, Lys, Met, Orn, Ser, and Tau relative to controls, whereas Ala, Cys, Gln, and Sar levels were higher in controls. ROC curves were then used to assess the potential value of these metabolites as LC biomarkers in Metaboanalyst [28] (Fig. 3) between LC patients with early-stage (stage I/II) and controls. To avoid overfitting, features were selected based upon overall ranks (AUC and T-statistic) and K-means (KM) clustering. This approach identified SM 42:4, Ser, Sar, Gln, and LPC 18:0 as a potential biomarker panel with an AUC of 0.965 (95% CI 0.900–1.000) (Additional file 1: Figure S2). As such, this analysis suggests this subset of metabolites may be useful for early-stage LC detection.

**Table 2:** Significantly altered metabolites in the different groups

No.	Metabolites	LB vs control	LA vs control	LB vs LA
1	Ala	#↓	△↓	–
2	Arg	–	△↑	–
3	Asn	#↑	–	△↑
4	Cer 38:1	△↑	△↑	–
5	Cer 42:2	△↑	△↑	–
6	Cer 43:1	△↑	△↑	–
7	Cer d18:1–22:2	–	#↑	–
8	Cit	–	–	#↑
9	Cys	#↓	–	–
10	FA 20:5	#↓	#↓	–
11	FA 22:4	–	△↑	–
12	FA 22:6	△↓	–	–
13	FA 26:0	#↑	–	–
14	GABA	–	#↓	△↑
15	Gln	△↓	△↓	–
16	Ile	–	△↓	–
17	Leu	–	△↓	–
18	LPC 14:0	△↓	△↓	–
19	LPC 15:0	△↓	#↓	–
20	LPC 16:0	△↓	△↓	–
21	LPC 16:1	△↓	#↓	–
22	LPC 17:0	△↓	△↓	–
23	LPC 17:1	△↓	#↓	–
24	LPC 18:0	△↓	△↓	–
25	LPC 18:1	△↓	#↓	–
26	LPC 20:0	△↓	△↓	–
27	LPC 20:1	△↓	△↓	–
28	LPC 20:2	–	△↓	△↑
29	LPC 22:0	–	#↓	–
30	LPC 22:4	–	#↑	–
31	LPC 22:5	–	#↑	–
32	LPC 22:6	#↓	–	–
33	LPC18:4	△↓	△↓	–
34	LPC 22:5	–	#↑	–
35	LPC 22:6	#↓	–	–
36	LPC18:4	△↓	△↓	–
37	LPE 16:0	#↓	–	–
38	LPE 20:2	–	#↓	△↑
39	LPE 20:4	–	△↑	–
40	LPE 20:5	△↓	△↓	–
41	Lys	#↑	△↑	–
42	Met	△↑	–	#↑
43	Orn	△↑	#↑	–
44	PC 29:1	–	–	#↓
45	PC 30:0	#↑	△↑	–
46	PC 30:1	△↑	△↑	–
47	PC 31:0	–	#↑	–
48	PC 32:0	△↑	△↑	–
49	PC 32:1	–	△↑	–
50	PC 33:1	–	#↑	–
51	PC 33:2	–	#↑	–
52	PC 34:0	–	△↑	–
53	PC 34:3	#↑	△↑	–
54	PC 34:4	–	#↑	–
55	PC 34:5	–	#↑	–
56	PC 35:1	#↑	△↑	–
57	PC 35:3	#↑	–	–
58	PC 36:0	△↑	–	–
59	PC 37:3	#↑	–	–
60	PC 37:4	#↑	–	–
61	PC 38:2	#↑	–	–
62	PC 38:5	△↑	–	–
63	PC 40:4	△↑	△↑	
64	PC 40:8	#↑	△↑	–
65	PC 42:2	–	#↓	–
66	PC 42:4	–	#↓	–
67	PC 42:5	–	△↑	–
68	PC 42:8	#↑	–	–
69	PC 43:5	△↑	△↑	–
70	PC33:3	–	#↑	–
71	PC38:0	–	△↑	–
72	PE16:0-16:0	#↑	#↑	–
73	Phe	–	△↓	–
74	Sar	△↓	△↓	–
75	Ser	△↑	–	#↑
76	SM 31:1	#↓	–	–
77	SM 32:2	–	#↑	–
78	SM 34:0	△↑	–	–
79	SM 34:1	#↑	△↑	–
80	SM 34:2	△↑	△↑	–
81	SM 35:0	#↑	△↑	△↓
82	SM 35:1	△↑	△↑	–
83	SM 36:1	△↑	△↑	–
84	SM 36:2	△↑	△↑	–
85	SM 36:3	–	△↑	–
86	SM 37:1	–	△↑	△↓
87	SM 37:2	–	#↑	–
88	SM 38:3	–	△↑	–
89	SM 41:1	–	△↑	–
90	SM 42:2	△↑	△↑	–
91	SM 42:4	△↑	△↑	–
92	SM 42:6	–	△↑	–
93	SM 43:1	#↓	–	–
94	SM 43:2	#↑	△↑	–
95	SM 35:3	△↑	△↑	–
96	TAG 50:0	–	#↑	△↓
97	TAG 52:3	#↓	–	–
98	TAG 54:1	#↓	–	–
99	TAG 54:2	△↓	#↓	–
100	TAG 54:3	#↓	–	–
101	TAG 54:4	#↓	–	–
102	Tau	#↑	#↑	–
103	Thr	–	△↓	△↑
104	Val	–	#↓	△↑

Following surgery, lower levels of Ala, GABA, Gln, Ile, Leu, Phe, Sar, Thr, Val, LPC, and LPE were detected in samples from the LA group relative to controls, whereas levels of Arg, Orn, Tau, Cer, SM, and PC were higher in the LA group relative to controls. Furthermore, significant changes in the levels of Asn, Cit, GABA, LPC 20:2, LPE 20:2, Met, PC 29:1, Ser, SM 35:0, SM 37:1, TAG 50:0, Thr, and SM 37:1 were detected when comparing pre- and post-surgery samples (Table 2).

### Metabolic pathway analysis

**Fig. 4:**
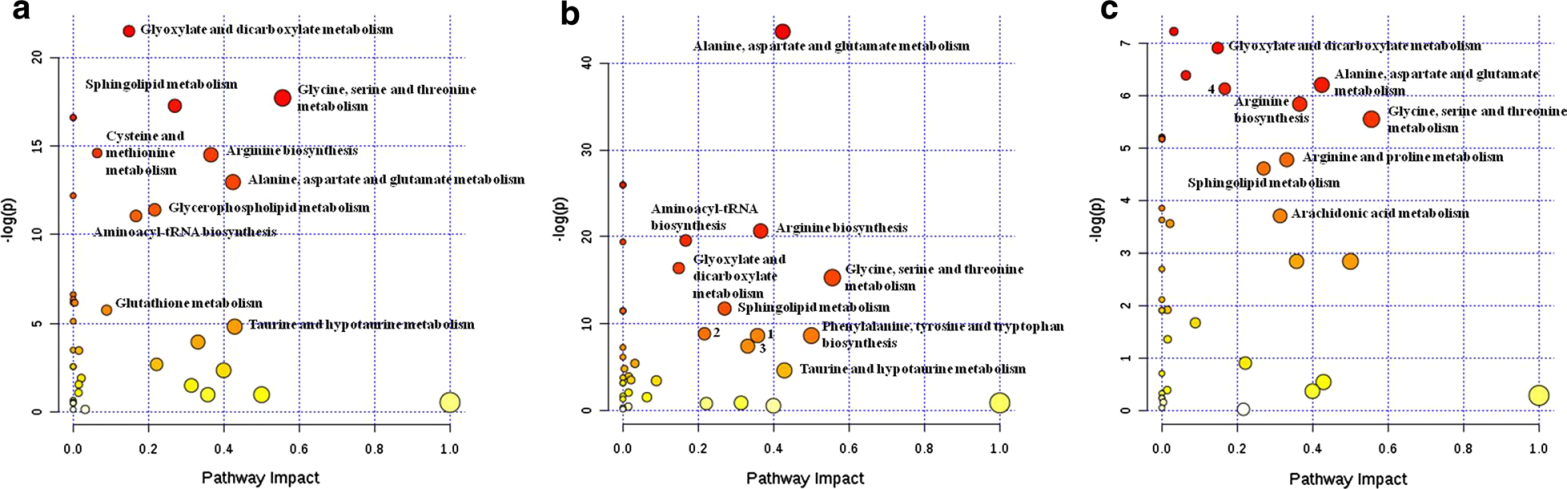
Significant differences in specific metabolic pathways were detected between the control, LA, and LB groups. Node size corresponds to the degree of impact on the indicated pathway, while node color corresponds to the associated *P*-value derived from a pathway enrichment analysis. **a** LB vs control; **b** LA vs control; **c** LB vs LA. 1: phenylalanine metabolism; 2: glycerophospholipid metabolism; 3: arginine and proline metabolism; 4: aminoacyl-tRNA biosynthesis

We next explored the potential pathways involved in regulating the differentially abundant metabolites detected in the above analysis using MetaboAnalyst, with those pathways that had low *P*-values and high pathway impacts being shown in Fig. 4.

Prior to surgery, LC-specific metabolites were primarily associated with the following metabolic pathways: Glycine, serine and threonine metabolism, Sphingolipid metabolism, Glycerophospholipid metabolism, Taurine and hypotaurine metabolism, Alanine, aspartate and glutamate metabolism, Arginine biosynthesis, Arginine and proline metabolism, Aminoacyl-tRNA biosynthesis, and Glyoxylate and dicarboxylate metabolism (Fig. 4a).

Following surgery, metabolites were primarily associated with: glycine, serine and threonine metabolism, alanine, aspartate and glutamate metabolism, sphingolipid metabolism, arginine biosynthesis, arginine and proline metabolism, arachidonic acid metabolism, aminoacyl-tRNA biosynthesis, and glyoxylate and dicarboxylate metabolism (Fig. 4b). In addition, 11 metabolic pathways were identified when comparing post-surgery LC patients to controls (Fig. 4c). For full details of affected pathways, see Additional file 1: Table S2.

### The prognostic relevance of CTCs and metabolic signatures in LC

Upon follow-up, 14 patients exhibited recurrent disease (SD + PD), with CTC numbers being increased in 13 of these patients following surgery. In contrast, 31 patients with non-recurrent disease (PR + CR), with CTC numbers being reduced in 29 of these patients following surgery (Additional file 1: Table S3). This suggests that CTC trends following surgery are consistent with patient prognosis.

**Fig. 5:**
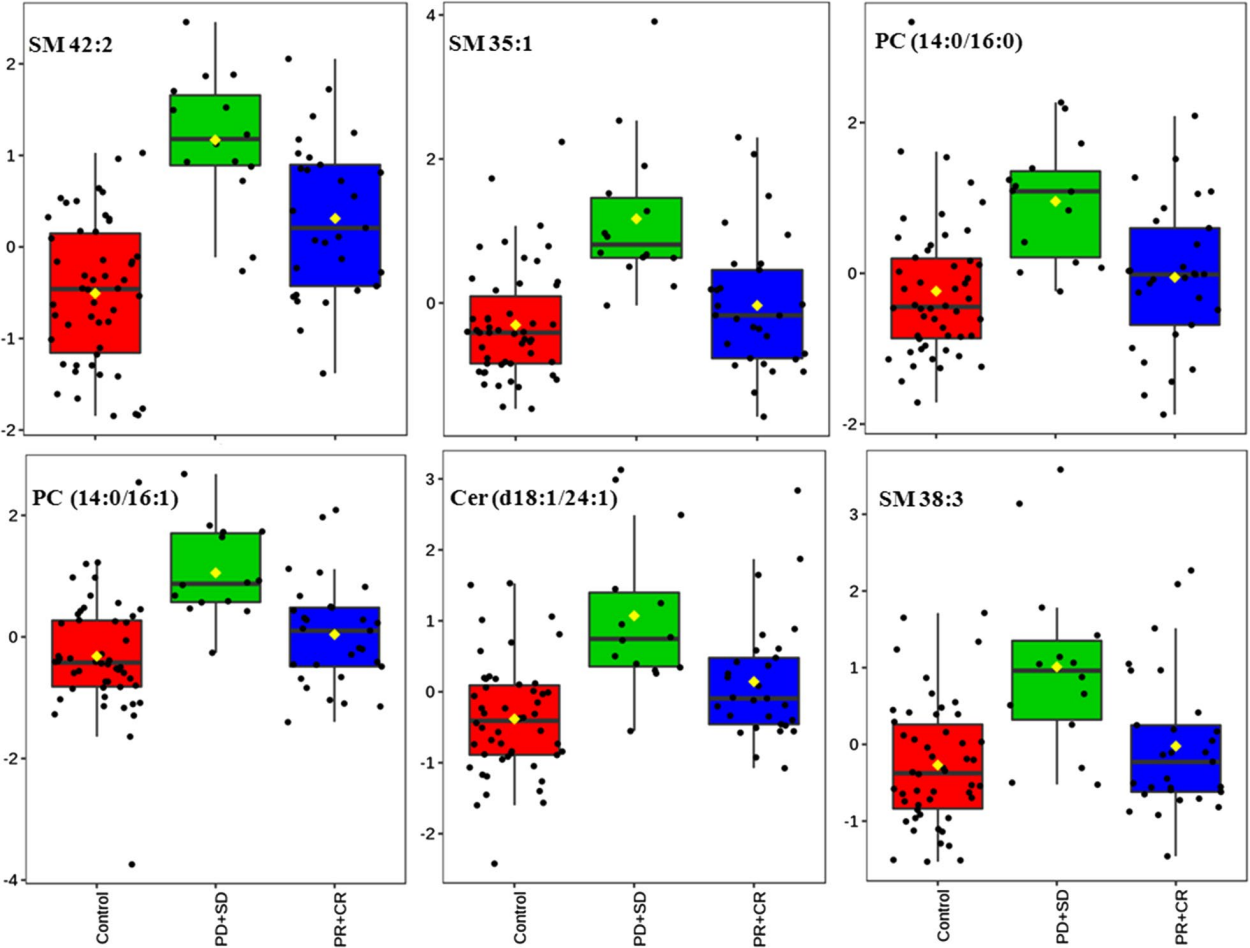
Box plots corresponding to levels of the six indicated metabolites in controls, patients with non-recurrent disease, and patients with recurrent disease. Non-recurrent patients: PR + CR group; recurrent patients: SD + PD group

Among the metabolites related to relapse, 25 metabolites were found to be significant increased in recurrent patients after surgery compared with nonrecurrent patients and the controls based on ANOVA analysis. Binary logistic regression was introduced for the early prediction of LC recurrence to analyze 25 metabolites. With respect to these metabolites, levels of SM 42:2, SM 35:1, PC 30:0, PC 30:1, Cer 42:2, and SM 38:3 were found to be present at significantly higher levels post-surgery in patients that suffered from LC recurrence relative to patients with non-recurrent disease or controls(Fig. 5) and showed significant changes (*P* < 0.05) from binary logistic regression. This therefore suggests that this metabolite panel may offer prognostic value as a means of predicting the likelihood of LC recurrence in patients undergoing surgery.

## Discussion

In the present study, we explored the relationship between CTCs, metabolic signatures, and LC patient post-surgical outcomes. We were able to detect CTCs in 66.7% of analyzed patients (34/51) prior to surgery. We observed surgery-related changes in CTC levels consistent with observed clinical outcomes (RECIST standard). Previous work suggests that CTCs may be valuable as diagnostic biomarkers of LC or for the identification of patients likely to benefit from specific treatments [32, 33]. Owing to the rarity of these cells and their non-epithelial characteristics, however, detecting CTCs in LC patients remains challenging. The previously reported CTC detection approach employed in the present study was nonetheless able to offer satisfactory clinical utility in LC [26].

Early LC diagnosis is essential, as patients with early-stage LC have a much better prognosis relative to patients with advanced disease [34, 35]. The identification of additional sensitive and specific biomarkers of LC is essential to facilitate the better detection and management of early-stage disease. Certain lipid signatures and other metabolites have been reported to be well-suited to differentiating between LC patients and healthy controls or individuals with non-malignant lung disease [36–39]. Metabolites in these signatures included choline-containing phospholipids, with alterations in precursor serum lysophosphatidylcholine levels thus having the potential to serve as valuable cancer biomarkers given that the metabolism of phospholipids such as phosphatidylcholine can be significantly disrupted in tumor cells [40]. We identified 14 LPC classes that were decreased in LC patient serum relative to controls, in line with previous research [38, 41, 42]. LPC acyltransferases (LPCATs) can convert LPC to PC in lung surfactant, which can lower surface tension and thereby facilitate respiration. LPCAT dysfunction is a hallmark of lung diseases, thus resulting in significant changes in LPC levels in affected individuals. Interestingly, we found that relative to controls, the levels of most measured PC classes were increased in LC patients prior to surgery. Chen et al. [43] detected significantly elevated levels of both monounsaturated and saturated PCs in LC patients, whereas these patients had lower polyunsaturated PC levels. Yet another study found that PC levels were elevated in LC patient serum [44]. Such elevated PC levels may be due to an increased need for these membrane phospholipids in cancer cells, given that they are primary components of the cell membrane [45, 46].

We observed elevated levels of sphingolipids such as ceramide (Cer) and sphingomyelin (SM) in LC patients relative to controls both pre- and post-surgery (Table 2). Sphingolipids are key bioactive molecules that can facilitate intracellular signaling and can control the growth and survival of tumor cells [47]. Cer is also thought to function as a pro-apoptotic lipid, the levels of which are increased in response to stress, thereby suppressing tumor growth [48]. Both chemotherapy and cytotoxic stimulation can promote endogenous Cer accumulation. Reduced amounts of Cer have been detected in both human colon cancer tissue [49] and ovarian tumors [50]. Despite these findings, other analyses have revealed that certain Cer species are in fact upregulated in cancer. For example, higher levels of long-chain ceramides have been detected in malignant breast cancer tissues relative to benign tissue controls [51]. This is consistent with our findings, suggesting that certain Cer species play specific roles in tumors. SM is a primary sphingolipid type found in cell membranes, and it can be converted into Cer by sphingomyelinase. Studies have explored the chemotherapeutic and chemopreventive potential of this pathway [52]. In one study, sphingomyelin hydrolysis and consequent disruption of ceramide synthesis were found to be associated with reduced NSCLC growth and enhanced anti-tumor immunity [53, 54].

We employed a rapid approach to sensitively and specifically detecting and quantifying the levels of 34 amino acids without derivatization in this study. Amino acids play central roles in metabolic networks, and have previously been shown to be of value for discriminating between LC patients and healthy controls [55–58]. The amino acid signatures detected in the present study were not identical to those in prior studies, potentially due to differences in population ethnicity, tumor stages, or LC histologic types in these studies [55–58]. However, the differentially abundant amino acid levels identified in this study have the potential to be used for early-stage LC diagnosis.

Surgery remains the best treatment option for LC patients with early-stage (stage I/II) disease, as it is associated with a significant increase in long-term patient survival [4, 5]. In a PLS-DA analysis, we found that metabolic shifts were evident in LC patients and controls (Fig. 3). We further employed pathway analyses in an effort to identify LC-related signaling pathways associated with the observed metabolic changes in our samples. These analyses revealed that these differentially abundant metabolites were associated with host responses to LC (Fig. 4a). In total, 9 and 11 differentially impacted metabolic pathways were identified in LC patients relative to controls before and after surgery, respectively (Fig. 4). A total of 8 metabolic pathways in LC patients with impacted by surgical intervention (Fig. 4b). The most significantly affected of these pathways included, the glycine, serine, and threonine metabolism pathways. Serine can be utilized for the synthesis of intermediates in the glycine, serine and threonine metabolism, protein, sphingolipid, purine and pyrimidine pathways [59]. Increased serine biosynthesis has been shown to drive cancer cell proliferation and to increase transformation rates in normal mammary epithelial cells [60]. Serine synthesis reportedly accounts for roughly half of total TCA cycle replenishment [59]. The upregulation of intermediates in the glycine, serine, and threonine metabolism pathways may thus be strongly correlated with increased glycolysis and of TCA cycle intermediate replenishment. In addition, we found that other amino acid-related pathways were associated with surgery in LC patients, including the Arginine biosynthesis, alanine, aspartate and glutamate metabolism, and Arginine and proline metabolism pathways.

We identified six different metabolites that were present at significantly higher levels in patients that went on to suffer from LC recurrence relative to patients with non-recurrent disease, including SM 42:2, SM 35:1, PC (16:0/14:0), PC (14:0/16:1), Cer (d18:1/24:1), and SM 38:3 (SD + PD group vs. PR + CR group, *P* < 0.05). This suggests that this biomarker combination may be of value for LC diagnosis and or prediction of LC recurrence in patients undergoing surgery (Fig. 5).

There are several limitations to the present analysis that must be considered. For one, this study was of a relatively small LC patient population, and future large-scale studies will be essential to draw any definitive conclusions regarding metabolic profiles associated with LC patient outcomes. In addition, additional assays are necessary to more fully explore and validate the surgery-associated metabolic changes detected in this study in LC patients, offering more insight into surgery-related pathophysiology. In addition, at present no correlation has been detected between CTCs and metabolic signatures. Future studies conducting single-cell metabolic profiling of these CTCs have the potential to offer direct functional insights into tumor cell metabolism in individual patients, facilitating a more direct understanding of the link between cancer cell genotype and metabolomics phenotype [61].

## Conclusions

In summary, our study demonstrates that LC patients had significantly higher CTC counts relative to healthy controls and changes of CTC counts were associated with clinical outcomes following surgery. Moreover, six metabolites were defined as the combinational biomarkers to recognize recurrence and nonrecurrence based on target metabolomics. Based on these essential findings, we suggest that CTC detection and plasma metabolite profiling may be an effective means of diagnosing early-stage LC and identifying patients at risk for disease recurrence.

## Supplementary information

**Additional file 1: Table S1.** All the annotated lipids in current study. **Table S2.** List of all the changed pathways among three groups based on metabolic pathway analysis. **Table S3.** Patients information and their prognosis status. **Figure S1.** Validation of PLS-DA model using permutation test. **Figure S2.** ROC curves for five metabolites (SM 42:4, Ser, Sar, Gln and LPC 18:0) to discriminate lung cancer patients from controls.

## Data Availability

All data generated or analyzed during this study are included in this article and its additional files.

## Abbreviations

LC: Lung cancer
NE-FISH: Negative enrichment-fluorescence in situ hybridization
CTCs: Circulating tumor cells
ctDNA: Circulating tumor DNA
CEA: Carcinoembryonic antigen
AFP: Alpha-fetoprotein
NSCLC: Non-small cell lung cancer
SCLC: Small cell lung cancer
UPLC/MS: Ultra-performance liquid chromatography/mass spectrometry
UPLC-QTOF/MS: Ultra-performance liquid chromatography with quadrupole time-of-flight mass spectrometry
Gly: Glycine
β-Ala: β-Alanine
Sar: Sarcosine
Ala: l-Alanine
Ser: l-Serine
Pro: l-Proline
Val: l-Valine
Thr: l-Threonine
Cys: l-Cysteine
Tau: Taurine
Leu: l-Leucine
GABA: γ-Aminobutyric acid
Ile: l-Isoleucine
t-Hyp: Trans-4-hydroxy-l-proline
Asn: l-Asparagine
Asp: l-Aspartic acid
Orn: l-Ornithine
Lys: l-Lysine
Glu: l-Glutamic acid
Met: l-Methionine
Phe: l-Phenylalanine
Arg: l-Arginine
Cit: l-Citrulline
Tyr: l-Tyrosine
Trp: l-Tryptophan
FA: Folic acid
His: l-Histidine
Gln: Glutamine
LPC: Lysophosphatidylcholine
PC: Phosphatidylcholine
LPE: Lysophosphatidylethanolamine
PE: Phosphatidylethanolamine
SM: Sphingomyelin
Cer: Ceramide
TAG: Triacylglycerol
IS: Internal standard
RT: Room temperature
CR: Complete response
SD: Stable disease
PR: Partial response
PD: Progressive disease
CEP: Chromosome centromere probe
ROC: Receiver operating characteristic
PLS-DA: Partial least squares discriminant analysis
LA: LC patients after surgery
LB: LC patients before surgery
LPCATs: LPC acyltransferases
RECIST: Response Evaluation Criteria in Solid Tumors
