# Supplementary material for: Clinical significance of circulating tumor cells and metabolic signatures in lung cancer after surgical removal
Source: J Transl Med. 2020 Jun 17;18:243. doi: 10.1186/s12967-020-02401-0 (PMC7301449; doi:10.1186/s12967-020-02401-0)
Supplement: Supplementary file 1 — Additional file 1: Table S1. All the annotated lipids in current study. Table S2. List of all the changed pathways among three groups based on metabolic pathway analysis. Table S3. Patients information and their prognosis status. Figure S1. Validation of PLS-DA model using permutation test. Figure S2. ROC curves for five metabolites (SM 42:4, Ser, Sar, Gln and LPC 18:0) to discriminate lung cancer patients from controls. [file 12967_2020_2401_MOESM1_ESM.docx]

**Clinical significance of circulating tumor cells and metabolic signatures in lung cancer after surgical removal**

Dawei Yang^1^, Xiaofang Yang^2^, Yang Li^1^, Peige Zhao^3^, Rao Fu^1^, Tianying Ren^1^, Ping Hu^1^, Yaping Wu^1^, Hongjun Yang^4*^, Na Guo^2*^

1. Zhong Yuan Academy of Biological Medicine, Liaocheng People’s Hospital, Liaocheng 252000, PR China
2. Experimental Research Center, China Academy of Chinese Medical Sciences, Beijing 100700, PR China
3. Department of Respiratory Medicine, Liaocheng People's Hospital, Liaocheng, 252000, PR China
4. Institute of Chinese Materia Medica, China Academy of Chinese Medical Sciences, Beijing 100700, PR China

**Correspondence:**

Prof. Na Guo

Experimental Research Center, China Academy of Chinese Medical Sciences, Beijing 100700, PR China

Tel: 86-10-64014411; E-mail: guona5246@126.com;

Prof. Hongjun Yang

Institute of Chinese Materia Medica, China Academy of Chinese Medical Sciences, Dongzhimen Nanxiao Road 16, Dongcheng District, Beijing 100700, China.

Tel: 86-10-64032656; E-mail address: [hongjun0420@vip.sina.com](mailto:hongjun0420@vip.sina.com)

Table S1 All the annotated lipids in current study.

| Compounds | Precursor Ion | Product Ion | Cone(V) | Collision (V) |
| --- | --- | --- | --- | --- |
| LPC 16:2 | 492.3 | 184.1 | 10 | 30 |
| LPC 14:0* | 468.3 | 184.1 | 10 | 30 |
| LPC 18:3* | 518.3 | 184.1 | 10 | 30 |
| LPC 20:5* | 542.3 | 184.1 | 10 | 30 |
| LPC 16:1* | 494.3 | 184.1 | 10 | 30 |
| LPC 15:0* | 482.3 | 184.1 | 10 | 30 |
| LPE 18:2* | 478.3 | 337.3 | 30 | 20 |
| LPE 22:6* | 526.3 | 385.3 | 30 | 20 |
| LPC 18:2* | 520.3 | 184.1 | 10 | 30 |
| LPE 20:4* | 502.3 | 361.3 | 30 | 20 |
| LPC 22:6* | 568.3 | 184.1 | 10 | 30 |
| LPC 20:4* | 544.3 | 184.1 | 10 | 30 |
| LPC 17:1* | 508.3 | 184.1 | 10 | 30 |
| LPE 16:0* | 454.3 | 313.3 | 30 | 20 |
| LPC 16:0* | 496.3 | 184.1 | 10 | 30 |
| LPE 20:5 | 500.3 | 359.3 | 30 | 20 |
| LPE 20:2 | 506.3 | 365.2 | 30 | 20 |
| LPC18:4* | 516.4 | 184.1 | 10 | 30 |
| LPC 20:2 | 548.4 | 184.1 | 10 | 30 |
| LPC 20:3* | 546.4 | 184.1 | 10 | 30 |
| LPC 22:5* | 570.4 | 184.1 | 10 | 30 |
| LPE 18:1* | 480.3 | 339.3 | 30 | 20 |
| LPC 18:1* | 522.4 | 184.1 | 10 | 30 |
| LPC 22:4* | 572.4 | 184.1 | 10 | 30 |
| LPC 17:0* | 510.4 | 184.1 | 10 | 30 |
| LPE 20:1 | 508.3 | 367.2 | 30 | 20 |
| LPC 17:2 | 506.3 | 184.1 | 10 | 30 |
| LPC 18:0* | 524.4 | 184.1 | 10 | 30 |
| LPE 18:0* | 482.3 | 341.3 | 30 | 20 |
| LPC 20:1* | 550.4 | 184.1 | 10 | 30 |
| LPC 19:0^#^ | 538.4 | 184.1 | 10 | 30 |
| LPC 20:0* | 552.4 | 184.1 | 10 | 30 |
| LPC 22:1* | 578.4 | 184.1 | 10 | 30 |
| LPC 21:0 | 566.4 | 184.1 | 10 | 30 |
| LPC 22:0* | 580.4 | 184.1 | 10 | 30 |
| LPC 24:1* | 606.5 | 184.1 | 10 | 30 |
| PE(12:0/13:0)^#^ | 594.4 | 453.4 | 31 | 50 |
| SM(d18:1/12:0)^#^ | 647.5 | 184.1 | 50 | 30 |
| SM(d16:1/16:1) * | 673.5 | 184.1 | 50 | 30 |
| LPC 24:0* | 608.5 | 184.1 | 10 | 30 |
| SM(d16:0/17:1) * | 661.5 | 184.1 | 50 | 30 |
| SM(d18:1/15:1) * | 687.5 | 184.1 | 50 | 30 |
| PC(18:3/16:2) * | 752.5 | 184.1 | 40 | 15 |
| PC(20:5/22:6) * | 852.6 | 184.1 | 40 | 15 |
| SM(d16:0/16:1) * | 675.5 | 184.1 | 50 | 30 |
| PC(12:0/16:0) * | 678.5 | 184.1 | 40 | 15 |
| PC(14:0/22:6) * | 778.5 | 184.1 | 40 | 15 |
| SM(d16:0/18:2) * | 701.6 | 184.1 | 50 | 30 |
| PC(20:4/22:6) * | 854.6 | 184.1 | 40 | 15 |
| PC(18:2/20:5) * | 804.6 | 184.1 | 40 | 15 |
| PC(16:1/18:3) * | 754.5 | 184.1 | 40 | 15 |
| PC(16:1/16:1) * | 730.5 | 184.1 | 40 | 15 |
| SM(d16:0/17:0) * | 691.6 | 184.1 | 50 | 30 |
| SM(d16:0/20:3) * | 727.6 | 184.1 | 50 | 30 |
| SM(d16:0/17:1) * | 689.6 | 184.1 | 50 | 30 |
| PC(10:0/19:1) | 690.5 | 184.1 | 40 | 15 |
| PC(15:0/22:6)* | 792.6 | 184.1 | 40 | 15 |
| PC(20:3/22:6) * | 856.6 | 184.1 | 40 | 15 |
| PC(18:2/22:6) | 830.6 | 184.1 | 40 | 15 |
| PC(16:0/20:5) * | 780.6 | 184.1 | 40 | 15 |
| SM(d18:1/17:1) * | 715.6 | 184.1 | 50 | 30 |
| PC(16:0/18:3) * | 756.6 | 184.1 | 40 | 15 |
| SM(d20:0/22:6) | 805.6 | 184.1 | 50 | 30 |
| PC(15:0/20:4) * | 768.6 | 184.1 | 40 | 15 |
| PC(15:0/18:2) * | 744.6 | 184.1 | 40 | 15 |
| PC(16:1/18:2) * | 756.6 | 184.1 | 40 | 15 |
| SM(d16:0/18:1) * | 703.6 | 184.1 | 50 | 30 |
| PC(14:0/16:1) * | 704.5 | 184.1 | 40 | 15 |
| PE(16:1 /18:2) * | 714.5 | 573.5 | 31 | 50 |
| SM(d20:0/22:5) | 807.6 | 184.1 | 50 | 30 |
| PE(16:0 /20:4) * | 740.5 | 599.5 | 31 | 50 |
| PC(17:1/18:2) * | 770.6 | 184.1 | 40 | 15 |
| PC(16:0/16:1) * | 732.6 | 184.1 | 40 | 15 |
| PC(18:1/22:6) * | 832.6 | 184.1 | 40 | 15 |
| SM(d16:0/20:2) * | 729.6 | 184.1 | 50 | 30 |
| PE(16:0 /22:6) * | 764.5 | 623.5 | 31 | 50 |
| PC(20:2/22:6) | 858.6 | 184.1 | 40 | 15 |
| SM(d16:0/22:3) * | 755.6 | 184.1 | 50 | 30 |
| SM(d16:0/18:0) * | 705.6 | 184.1 | 50 | 30 |
| PC(12:0/18:2) * | 706.5 | 184.1 | 40 | 15 |
| PC(15:0/16:0) * | 720.6 | 184.1 | 40 | 15 |
| PC(16:0/17:1) * | 746.6 | 184.1 | 40 | 15 |
| PC(18:0/20:5) * | 808.6 | 184.1 | 40 | 15 |
| SM(d16:0/19:1) * | 717.6 | 184.1 | 50 | 30 |
| PE(16:1 /18:1) * | 716.5 | 575.5 | 31 | 50 |
| PE(16:0 /22:5) * | 766.5 | 625.5 | 31 | 50 |
| PE(16:0 /20:3) * | 742.5 | 601.5 | 31 | 50 |
| PC(17:0/20:4) * | 796.6 | 184.1 | 40 | 15 |
| PC(16:1/19:1) * | 772.6 | 184.1 | 40 | 15 |
| PC(16:0/16:0) * | 734.6 | 184.1 | 40 | 15 |
| PC(15:1/18:2) * | 742.5 | 184.1 | 40 | 15 |
| SM(d18:1/19:1) * | 743.6 | 184.1 | 50 | 30 |
| PC(18:0/22:6) * | 834.6 | 184.1 | 40 | 15 |
| SM(d16:0/20:1) * | 731.6 | 184.1 | 50 | 30 |
| SM(d18:2/17:1) * | 713.6 | 184.1 | 50 | 30 |
| PC(20:1/22:6) * | 860.6 | 184.1 | 40 | 15 |
| PE(17:0 /18:2) * | 730.5 | 589.5 | 31 | 50 |
| Cer(d18:1/16:0) * | 538.5 | 264.3 | 20 | 30 |
| PC(18:2/19:1) * | 798.6 | 184.1 | 40 | 15 |
| PE(16:0/16:0) * | 692.5 | 551.5 | 31 | 50 |
| PC(19:1/20:4) * | 822.6 | 184.1 | 40 | 15 |
| PC(18:2/22:3) * | 836.6 | 184.1 | 40 | 15 |
| PE(18:0 /22:5) * | 794.6 | 653.6 | 31 | 50 |
| SM(d20:0/22:4) | 809.7 | 184.1 | 50 | 30 |
| PE(16:0 /18:1) * | 718.5 | 577.5 | 31 | 50 |
| SM(d16:0/19:0) | 719.6 | 184.1 | 50 | 30 |
| PE(18:0 /20:4) * | 768.6 | 627.5 | 31 | 50 |
| PC(20:0/22:6) * | 862.6 | 184.1 | 40 | 15 |
| PE(18:1 /18:1) * | 744.6 | 603.5 | 31 | 50 |
| PC(16:0/19:1) * | 774.6 | 184.1 | 40 | 15 |
| Cer(d18:1/17:0)^#^ | 552.5 | 264.3 | 20 | 30 |
| SM(d18:1/19:0) * | 745.6 | 184.1 | 50 | 30 |
| PC(19:0/20:4) * | 824.6 | 184.1 | 40 | 15 |
| PC(18:2/19:0) * | 800.6 | 184.1 | 40 | 15 |
| PC(16:0/18:0) * | 762.6 | 184.1 | 40 | 15 |
| PE(18:0 /20:3) * | 770.6 | 629.6 | 31 | 50 |
| PC(18:0/22:4) * | 838.6 | 184.1 | 40 | 15 |
| PC(18:0/18:1) * | 788.6 | 184.1 | 40 | 15 |
| PC(19:0/22:6) * | 848.6 | 184.1 | 40 | 15 |
| PE(20:0 /20:4) * | 796.6 | 655.6 | 31 | 50 |
| PC(18:2/20:0) * | 814.6 | 184.1 | 40 | 15 |
| Cer(d18:1/18:0) * | 566.6 | 264.3 | 20 | 30 |
| PC 42:5-2 | 864.6 | 184.1 | 40 | 15 |
| PC(16:2/24:1) | 840.6 | 184.1 | 40 | 15 |
| PE(18:0 /18:1) * | 746.6 | 605.6 | 31 | 50 |
| PC(20:0/22:4) * | 866.7 | 184.1 | 40 | 15 |
| SM(d20:0/19:1) * | 773.7 | 184.1 | 50 | 30 |
| PC(16:0/20:0) * | 790.6 | 184.1 | 40 | 15 |
| PC(16:0/22:1) * | 816.6 | 184.1 | 40 | 15 |
| PC(20:5/23:0) | 878.7 | 184.1 | 40 | 15 |
| SM(d18:1/24:1) * | 813.7 | 184.1 | 50 | 30 |
| Cer(d18:1/20:0) * | 594.6 | 264.3 | 20 | 30 |
| PC(16:2/26:1) | 868.7 | 184.1 | 40 | 15 |
| PC(18:1/22:1) * | 842.7 | 184.1 | 40 | 15 |
| Cer(d18:2/22:0) * | 620.6 | 262.2 | 20 | 30 |
| Cer(d18:2/24:1) * | 646.6 | 262.2 | 20 | 30 |
| SM(d16:1/25:0) | 801.7 | 184.1 | 50 | 30 |
| PC(19:0/19:0)^#^ | 818.6 | 184.1 | 40 | 15 |
| Cer(d16:1/24:1)* | 620.6 | 236.2 | 20 | 30 |
| SM(d18:2/25:0) | 827.7 | 184.1 | 50 | 30 |
| SMd16:0/26:1)* | 815.7 | 184.1 | 50 | 30 |
| Cer(d18:1/24:1)* | 648.6 | 264.3 | 20 | 30 |
| Cer(d18:1/22:0)* | 622.6 | 264.3 | 20 | 30 |
| PC(18:0/24:2)* | 870.7 | 184.1 | 40 | 15 |
| SM(d18:1/25:0) | 829.7 | 184.1 | 50 | 30 |
| Cer(d18:1/22:2)* | 618.6 | 264.3 | 20 | 30 |
| Cer(d18:1/24:0)* | 650.6 | 264.3 | 20 | 30 |
| Cer(d18:1/25:0) | 664.7 | 264.3 | 20 | 30 |
| Cer(d18:1/26:1)* | 676.7 | 264.3 | 20 | 30 |
| TG(18:4/16:0/18:2)* | 868.6 | 597.4 | 35 | 20 |
| TG(16:0/14:0/18:3)* | 818.7 | 547.4 | 35 | 20 |
| TG(16:0/16:1/18:3) | 844.6 | 545.4 | 35 | 20 |
| TG(16:0/18:2/18:3) | 870.6 | 597.4 | 35 | 20 |
| TG(18:1/18:2/18:3) | 896.6 | 625.4 | 35 | 20 |
| TG(16:1/14:0/18:1) | 820.7 | 549.4 | 35 | 20 |
| TG(16:0/16:2/18:1)* | 846.6 | 575.4 | 35 | 20 |
| TG(15:0/15:0/15:0)^#^ | 782.55 | 523.4 | 35 | 20 |
| TG(16:0/18:2/18:2) | 872.6 | 573.4 | 35 | 20 |
| TG(18:1/18:2/18:2) | 898.6 | 627.4 | 35 | 20 |
| TG(16:0/14:0/18:1) | 822.6 | 549.4 | 35 | 20 |
| TG(16:0/16:1/18:1) | 848.6 | 575.4 | 35 | 20 |
| TG(16:0/16:0/20:3)* | 874.6 | 603.4 | 35 | 20 |
| TG(16:0/18:1/20:3)* | 900.8 | 629.4 | 35 | 20 |
| TG(18:0/14:0/16:0)* | 824.6 | 551.4 | 35 | 20 |
| TG(16:0/16:0/18:1) | 850.6 | 577.4 | 35 | 20 |
| TG(18:0/16:0/18:2)* | 876.6 | 575.4 | 35 | 20 |
| TG(18:0/18:1/18:2) | 902.6 | 629.4 | 35 | 20 |
| TG(18:0/16:0/16:0) | 852.6 | 579.4 | 35 | 20 |
| TG(18:0/18:1/18:1) | 904.8 | 631.4 | 35 | 20 |
| TG(18:0/18:0/18:1) | 906.6 | 605.4 | 35 | 20 |
| FA14:1* | 225.2 | 225.2 | 10 | 10 |
| FA 16:2 | 251.2 | 251.2 | 10 | 10 |
| FA 20:5* | 301.2 | 301.2 | 10 | 10 |
| FA 14:0* | 227.2 | 227.2 | 10 | 10 |
| FA 18:3* | 277.2 | 277.2 | 10 | 10 |
| FA 22:6* | 327.2 | 327.2 | 10 | 10 |
| FA 16:1* | 253.2 | 253.2 | 10 | 10 |
| FA 20:4* | 303.2 | 303.2 | 10 | 10 |
| FA15:0* | 241.2 | 241.2 | 10 | 10 |
| FA 18:2* | 279.2 | 279.2 | 10 | 10 |
| FA 17:1* | 267.2 | 267.2 | 10 | 10 |
| FA 22:5* | 329.2 | 329.2 | 10 | 10 |
| FA 20:3* | 305.2 | 305.2 | 10 | 10 |
| FA 16:0_d3^#^ | 258.3 | 258.3 | 10 | 10 |
| FA 16:0* | 255.2 | 255.2 | 10 | 10 |
| FA 22:4* | 331.3 | 331.3 | 10 | 10 |
| FA 18:1* | 281.2 | 281.2 | 10 | 10 |
| FA 20:2* | 307.3 | 307.3 | 10 | 10 |
| FA 17:0* | 269.2 | 269.2 | 10 | 10 |
| FA 18:0_d3^#^ | 286.3 | 286.3 | 10 | 10 |
| FA 18:0* | 283.3 | 283.3 | 10 | 10 |
| FA 19:0^#^ | 297.2 | 297.2 | 10 | 10 |
| FA 20:0* | 311.3 | 311.3 | 10 | 10 |
| FA 22:1* | 337.3 | 337.3 | 10 | 10 |
| FA 22:0* | 339.3 | 339.3 | 10 | 10 |
| FA 24:0* | 367.4 | 367.4 | 10 | 10 |
| FA 26:0* | 395.4 | 395.4 | 10 | 10 |

Table S2 List of all the changed pathways among three groups based on metabolic pathway analysis

|  | **Pathway** | **Raw *P*** | **FDR** | **Impact** |
| --- | --- | --- | --- | --- |
| LB vs Control | Glycine, serine and threonine metabolism | 2.02E-08 | 3.94E-07 | 0.55577 |
|  | Taurine and hypotaurine metabolism | 0.00801 | 0.016442 | 0.42857 |
|  | Alanine, aspartate and glutamate metabolism | 2.34E-06 | 1.01E-05 | 0.42388 |
|  | Arginine biosynthesis | 5.01E-07 | 2.44E-06 | 0.36548 |
|  | Arginine and proline metabolism | 0.019112 | 0.037268 | 0.33113 |
|  | Sphingolipid metabolism | 3.15E-08 | 3.99E-07 | 0.26978 |
|  | Glycerophospholipid metabolism | 1.12E-05 | 3.96E-05 | 0.21631 |
|  | Aminoacyl-tRNA biosynthesis | 1.58E-05 | 5.14E-05 | 0.16667 |
|  | Glyoxylate and dicarboxylate metabolism | 4.66E-10 | 1.82E-08 | 0.14815 |
| LB vs LA | Glycine, serine and threonine metabolism | 0.003882 | 0.018428 | 0.55577 |
|  | Alanine, aspartate and glutamate metabolism | 0.002018 | 0.016935 | 0.42388 |
|  | Arginine biosynthesis | 0.00291 | 0.018428 | 0.36548 |
|  | Arginine and proline metabolism | 0.008417 | 0.02525 | 0.33113 |
|  | Arachidonic acid metabolism | 0.024567 | 0.059883 | 0.3135 |
|  | Sphingolipid metabolism | 0.009938 | 0.027683 | 0.26978 |
|  | Aminoacyl-tRNA biosynthesis | 0.002171 | 0.016935 | 0.16667 |
|  | Glyoxylate and dicarboxylate metabolism | 0.000998 | 0.016935 | 0.14815 |
| LA vs Control | Glycine, serine and threonine metabolism | 2.17E-07 | 9.42E-07 | 0.55577 |
|  | Phenylalanine, tyrosine and tryptophan biosynthesis | 0.000172 | 0.000419 | 0.5 |
|  | Taurine and hypotaurine metabolism | 0.009415 | 0.016691 | 0.42857 |
|  | Alanine, aspartate and glutamate metabolism | 1.17E-19 | 4.55E-18 | 0.42388 |
|  | Arginine biosynthesis | 1.07E-09 | 8.31E-09 | 0.36548 |
|  | Phenylalanine metabolism | 0.000172 | 0.000419 | 0.35714 |
|  | Arginine and proline metabolism | 0.000578 | 0.001326 | 0.33113 |
|  | Sphingolipid metabolism | 7.65E-06 | 2.99E-05 | 0.26978 |
|  | Glycerophospholipid metabolism | 0.000139 | 0.000388 | 0.21631 |
|  | Aminoacyl-tRNA biosynthesis | 3.17E-09 | 2.06E-08 | 0.16667 |
|  | Glyoxylate and dicarboxylate metabolism | 7.29E-08 | 3.55E-07 | 0.14815 |

Table S3 Patients information and their prognosis status

| Patient ID | Histology | TNM stage | CTC | counts | Prognosis |
| --- | --- | --- | --- | --- | --- |
|  |  |  | Pre-surgery | Pos-surgery |  |
| 1 | Adenocarcinoma | T4N0M0 | 30 | 3 | PR+CR |
| 2 | Adenocarcinoma | T1aN0M0 | 9 | 4 | PR+CR |
| 3 | Adenocarcinoma | T1aN1M0 | 9 | 5 | PR+CR |
| 4 | Adenocarcinoma | T4N1M0 | 7 | 3 | PR+CR |
| 5 | Adenocarcinoma | T1aN0M0 | 7 | 4 | PR+CR |
| 6 | Adenocarcinoma | T1NxM0 | 6 | 0 | PR+CR |
| 7 | Adenocarcinoma | T1aN1M0 | 4 | 2 | PR+CR |
| 8 | Adenocarcinoma | T1aN3M0 | 4 | 2 | PR+CR |
| 9 | Squamous | T4N1M0 | 4 | 3 | PR+CR |
| 10 | Adenocarcinoma | T2aN1M0 | 3 | 0 | PR+CR |
| 11 | SCLC | T2aN1M0 | 3 | 0 | PR+CR |
| 12 | Adenocarcinoma | T1N3M0 | 3 | 2 | PR+CR |
| 13 | Adenocarcinoma | T1bN0M0 | 3 | 2 | PR+CR |
| 14 | SCLC | T1bN0M0 | 2 | 0 | PR+CR |
| 15 | Squamous | T2aN1M0 | 2 | 0 | PR+CR |
| 16 | Squamous | T2aN0M0 | 2 | 0 | PR+CR |
| 17 | Adenocarcinoma | T2aN1M0 | 1 | 0 | PR+CR |
| 18 | Adenocarcinoma | T4N1M0 | 1 | 0 | PR+CR |
| 19 | Squamous | T3N1M0 | 1 | 0 | PR+CR |
| 20 | Squamous | T2aN1M0 | 3 | 3 | PR+CR |
| 21 | Squamous | T2aN1M0 | 3 | 3 | PR+CR |
| 22 | Squamous | T3N1M0 | 1 | 1 | SD+PD |
| 23 | Squamous | T1bN0M0 | 1 | 1 | PR+CR |
| 24 | SCLC | T2aN3M1 | 1 | 1 | PR+CR |
| 25 | Adenocarcinoma | T1aN0M0 | 0 | 0 | PR+CR |
| 26 | Adenocarcinoma | T2aN1M0 | 0 | 0 | None |
| 27 | Adenocarcinoma | T1aN0M0 | 0 | 0 | PR+CR |
| 28 | Squamous | T1aN0M0 | 0 | 0 | PR+CR |
| 29 | Squamous | T1aN0M0 | 0 | 0 | PR+CR |
| 30 | Adenocarcinoma | T2aN0M0 | 0 | 0 | PR+CR |
| 31 | Adenocarcinoma | T1aN0M0 | 0 | 0 | PR+CR |
| 32 | Adenocarcinoma | T1bN0M0 | 0 | 45 | SD+PD |
| 33 | Adenocarcinoma | T3N3M0 | 3 | 21 | SD+PD |
| 34 | Adenocarcinoma | T3N0M0 | 3 | 8 | SD+PD |
| 35 | Squamous | T1aNxM0 | 3 | 6 | SD+PD |
| 36 | Squamous | T2aNxM1a | 0 | 5 | SD+PD |
| 37 | Squamous | T2aNxM0 | 1 | 5 | SD+PD |
| 38 | Squamous | T1bN0M0 | 3 | 5 | SD+PD |
| 39 | Adenocarcinoma | T1aN0M0 | 1 | 4 | SD+PD |
| 40 | Adenocarcinoma | T2bN0M0 | 3 | 4 | None |
| 41 | Adenocarcinoma | T1aN0M0 | 0 | 3 | SD+PD |
| 42 | Adenocarcinoma | T1bN0M0 | 2 | 3 | SD+PD |
| 43 | Adenocarcinoma | T1bN0M0 | 0 | 2 | SD+PD |
| 44 | Squamous | T2aN0M0 | 0 | 2 | SD+PD |
| 45 | Adenocarcinoma | T1bN0M0 | 0 | 2 | SD+PD |
| 46 | SCLC | T1aN0M0 | 0 | 2 | None |
| 47 | Adenocarcinoma | T1aN0M0 | 0 | 2 | None |
| 48 | Squamous | T2aN0M0 | 1 | 2 | PR+CR |
| 49 | Adenocarcinoma | T1bN1M0 | 1 | 2 | None |
| 50 | Adenocarcinoma | T1aN0M0 | 0 | 1 | None |
| 51 | Adenocarcinoma | T1aN0M0 | 0 | 1 | PR+CR |

None: loss of follow-up


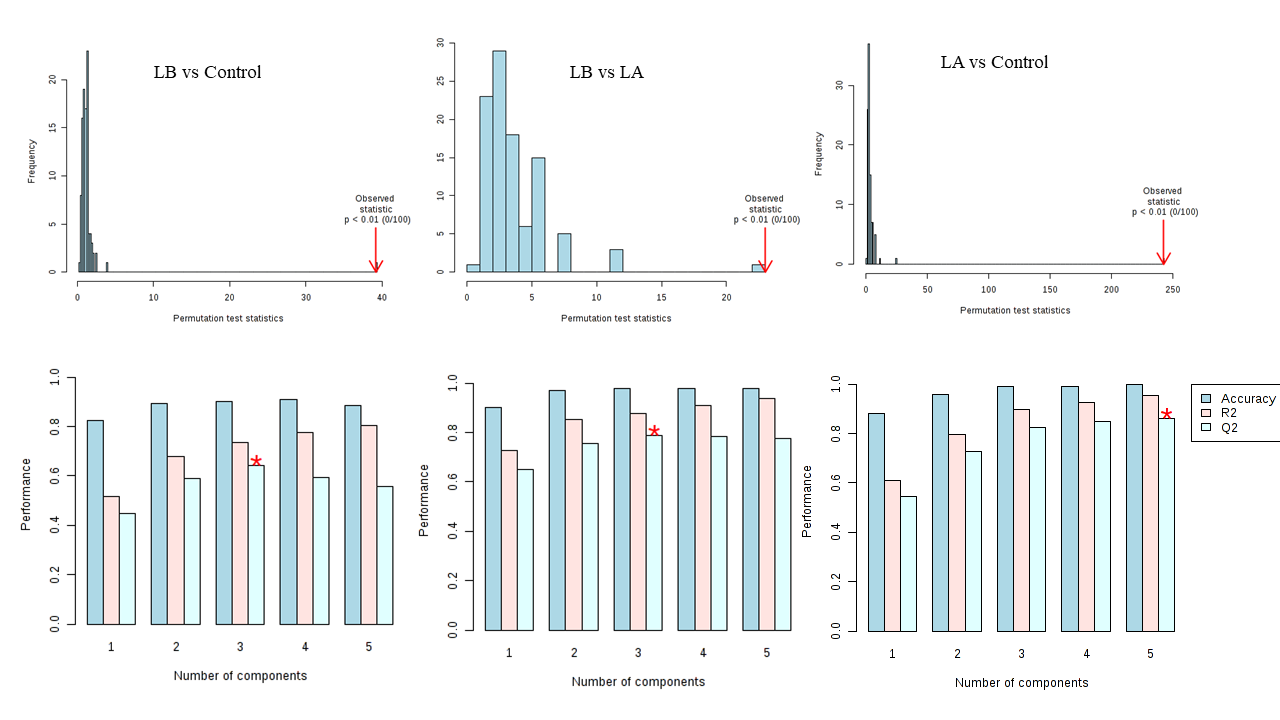


Figure S1 Validation of PLS-DA model using permutation test


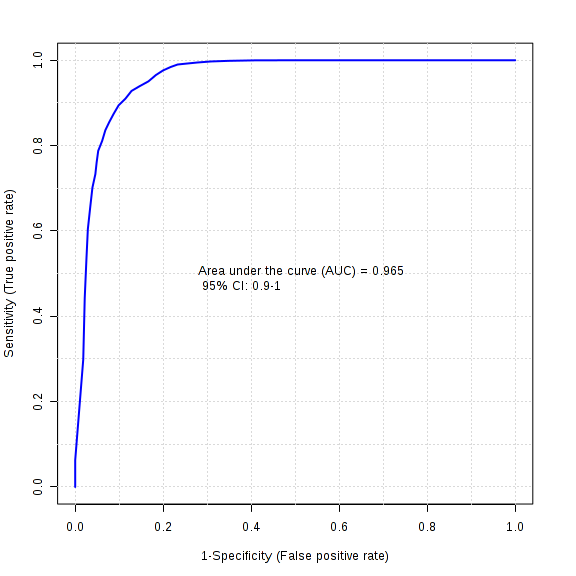


Figure S2 ROC curves for five metabolites (SM 42:4, Ser, Sar, Gln and LPC 18:0) to discriminate lung cancer patients from controls.
